# How Can Cities Adapt to a Multi-Disaster Environment? Empirical Research in Guangzhou (China)

**DOI:** 10.3390/ijerph15112453

**Published:** 2018-11-03

**Authors:** Yijun Shi, Guofang Zhai, Shutian Zhou, Yuwen Lu, Wei Chen, Hongbo Liu

**Affiliations:** 1 School of Architecture and Urban Planning, Nanjing University, Nanjing 210093, China; shutian_zhou@smail.nju.edu.cn (S.Z.); yuwen_lu@smail.nju.edu.cn (Y.L.); 2 School of Geography and Ocean Sciences, Nanjing University, Nanjing 210093, China; chenw.nju@gmail.com; 3 China Academy of Urban Planning and Design, Beijing 100044, China; hongboliu923@163.com

**Keywords:** disaster risk assessment, multi-disaster environment, risk level, urban safety strategy, Guangzhou City

## Abstract

Urban disaster risk assessment is the most basic and important part of urban safety development. Conducting disaster prevention and mitigation on the basis of urban disaster risk assessment requires an understanding of the relationship between the city and the natural environment. This enhances the city’s ability to withstand various types of disasters and achieves the development of a safe city. Based on a review of the existing literature, we propose a fuzzy comprehensive evaluation method for urban multi-disaster risk assessment. The multi-disaster risk assessment method includes the identification and screening of urban disasters, the assessment of individual disaster risk, and integrated urban disaster risks, the division of urban comprehensive disaster risks into several risk levels, and the determination of coping strategies. Taking Guangzhou (China) as an example, we determined the major disaster risks faced by Guangzhou, assessed the risks of individual disasters, and finally obtained the results of the comprehensive disaster risk of Guangzhou. Second, we analyzed the relationship between the disaster risk assessment and urban safety development, and proposed countermeasures and recommendations for the development of different disaster risk levels.

## 1. Introduction

Any sudden or calamitous event that endangers human life and property and affects living conditions is defined as a disaster. Disasters are mainly categorized as natural disasters (earthquakes, floods, typhoons, mudslides, etc.) and man-made disasters (fires, medical accidents, crimes, environmental pollution, etc.). As a result of ongoing population growth and the economic expansion, there has been an increasing trend in the frequency of disasters and disaster losses in urban areas. Disasters often result in serious consequences, especially in developing countries. [1] According to the United Nations Office for Disaster Reduction (UNISDR), in 2015 alone, nearly one billion people worldwide were affected by natural disasters [1,2]. In recent years, various types of disasters have occurred frequently around the world, such as the Indian Ocean tsunami in 2004, Hurricane Katrina in New Orleans in 2005, the tsunami in the Miyagi Prefecture in Japan in 2011, and the super-storm Sandy in New Jersey in 2012. These disasters have caused a devastating blow to people’s lives and productivity. The occurrence of various types of disasters is a serious threat to human survival and development. Social progress and the reduction of risk and disasters are the long-term goals of humankind. In 1981, the International Risk Association (SRA) was established to systematically conduct disaster risk analysis and research. In 1994, the first International Conference on Disaster Reduction of the United Nations adopted the Yokohama Statement and the Action Plan for Disaster Reduction to propose guidelines for a safer world. In 2005, the Second UN Conference on Global Disaster Reduction adopted the Hyogo Declaration [3], which identified disaster risk identification, evaluation, disaster risk monitoring, and early warning as the five priority areas for disaster reduction in the next 10 years. The International Strategy for Disaster Reduction adopted by the 60th UN General Assembly in 2005 further promoted the transformation of disaster risk research and management concepts. In 2008, the International Council for Science (ICSU) presented the Integrated Research on Disaster Risk (IRDR), focusing on natural and man-made environmental disaster risks [4]. In addition, in recent years, the Davos Forum in Switzerland has held a series of international disaster and risk conferences, making it one of the most important international conferences in the field of international disaster risk assessment and management [5]. These projects and conferences have promoted the expansion of natural disaster research. It has become an important trend to focus on and explore disaster risks. Risk assessment has become a necessary step and foundation for the scientific formulation of urban disaster reduction policies. A comprehensive understanding and correct assessment of the risks to human society as a result of disasters are required for effectively improving urban disaster prevention and mitigation and are also urgently needed for urban safety development [6,7].

China has a large population and a complex natural environment. It suffers the highest number of disasters and the most serious disasters. The estimated annual economic losses caused by disasters in China range from 150 billion CNY to 250 billion CNY [8]. Various types of disasters that have occurred in the past 10 years, such as the Wenchuan earthquake in 2008, the Yushu earthquake in 2010, the Zhouqu mudslide in 2010, the Shanghai Bund stampede in 2014, super typhoon Moranti in 2016, the Sichuan mountain collapse in 2017, and the torrential rains of 2018 have all caused serious losses to people’s lives and productivity. At present, China is in a period of rapid urbanization. An increasing number of people are moving into cities. On the one hand, this has resulted in the prosperity and development of cities. On the other hand, it has also caused large impacts on cities, making them more fragile. In the face of various natural disasters and man-made disasters, cities often exhibit great vulnerability, which becomes a bottleneck that restricts urban survival and sustainable development. Therefore, it is necessary to clearly understand the current situation of disaster risks faced by cities in the process of urbanization and to assess the disaster risks faced by cities. Countermeasures for urban adaptation to disaster risks have been proposed to improve a city’s ability to respond to disasters and achieve sustainable urban development [9]. The objective of this study is to explore the methods of urban disaster risk assessment and put forward strategies for urban adaptations to the disaster risk environment.

Our paper offers two main contributions. First, due to the large number of factors affecting disaster risk, this makes the characteristics of complexity and uncertainty between different disaster triggering factors. The fuzzy comprehensive evaluation method can better analyze the random ambiguity problem and is suitable for multi-index comprehensive evaluation. Therefore, in this paper, we construct a multi-disaster risk assessment method based on fuzzy comprehensive evaluation method. We use Guangzhou City as an example, determine the major disaster risks faced by Guangzhou City, assess the risks of individual disasters, and finally determine the comprehensive disaster risk of Guangzhou City. Second, based on the comprehensive assessment of the disaster risk in Guangzhou, we analyze the relationship between disaster risk assessment and urban safety development and propose countermeasures and recommendations for the development of different disaster risk levels.

The paper is organized as follows: in Section 2, we review the current indicators and methods for assessing disaster risk. In Section 3, we introduce the study area and methods used in this study. In Section 4, we use Guangzhou as an example and assess the disaster risks of five major disasters including floods, storm surges, earthquakes, geological disasters, and fires. Based on the assessment of different disasters, we present the results of the comprehensive disaster risk assessment of Guangzhou City. In Section 5, we analyze the relationship between disaster risk assessment and urban safety development and proposed countermeasures and recommendations for the development of different disaster risk levels. In Section 6, we draw the main conclusions.

## 2. Literature Review

Disaster risk assessment is a core component of disaster risk management and also an important bridge linking disaster risk analysis with urban vulnerability analysis [10]. Scholars have conducted research on disaster risk assessment from different aspects and have made considerable progress. In current research on disaster risk assessment, in addition to emphasizing the internal mechanism and risk assessment of the disaster, an increasing number of scholars have conducted comprehensive research on the human acceptance of risk levels from the perspectives of society, economy, and human behavior [11]. The research focus has gradually moved towards the research on socioeconomic security because risk assessment and risk management are linked and research on the impacts of disaster risk on the socioeconomic development is highly valued [12,13,14].

The global disaster research program has had an important impact on the establishment of a disaster risk assessment index system. The Disaster Risk Indicators (DRI) program, which was developed by the United Nations Development Program (UNDP) in collaboration with the United Nations Environment Programme (UNEP), has developed an index system for disaster risk. The Disaster Risk Hotspots program, jointly developed by Columbia University and the ProVention Alliance, establishes three risk assessment indicators for disaster-prone areas and compiles the assessment results into different levels of disaster risk maps [15,16]. The two research projects are conducted at the national scale and the focus of research is on natural disasters around the world. The results of these research projects provide ideas and tools for comprehensive risk research of urban disasters. The research on disaster risk project conducted by the Inter-American Development Bank (IADB) and Columbia University from 2003 to 2004 proposes an index system for evaluating disaster risk at the regional and city scales. These indicators have played an important role in promoting disaster risk assessment in cities and regions. Currently, the ProVention Alliance and UNDP are launching the Global Risk Identification Program (GRIP) [17], a global program for assessing, identifying, and analyzing disaster risks and losses. The objectives of the program are to provide important information for decision-making to reduce disaster risk, to help identify priority areas for risk reduction, to use more detail and in-depth information in disaster risk research, and to focus on risk inputs such as losses and cost-effectiveness in risk assessment.

Disaster risk assessment must have clear objectives, targets, factors, processes, and methods [18]. The traditional disaster risk assessment includes four steps: establishing a disaster assessment model, estimating risk loss, dividing risk levels, and creating risk maps [19,20]. At present, systematic research on disaster risk assessment has mainly focused on single disasters. The comprehensive assessment of multi-disaster events has only gradually attracted attention since the end of the 20th century and the research focus has gradually shifted from single disaster assessment to comprehensive assessment of multiple disasters. Multi-disaster risk assessment is a process of evaluating the comprehensive impact of regional development and the safety of residents and their property by considering multiple disasters with different impacts and different characteristics in one region. Multi-disaster risk assessments are generally based on single-hazard risk studies [6,18]. In existing research, there are two main approaches for applying single-disaster research to multi-disaster comprehensive research; the first method is based on combining the risk factors to obtain the comprehensive risk and comprehensive vulnerability [21]. The second method is to superimpose the risk results of the single disasters to obtain the risk of multiple disasters [22]. In a complex disaster, the method of simply summing up the risks of a single disaster lacks reliability. Therefore, the comprehensive analysis of multiple disasters is the key to multi-disaster assessment.

At present, the multi-disaster comprehensive assessment model with a certain representativeness includes the following: (1) The time between the disaster occurrence and the assessment is divided into a pre-assessment of the site before the disaster occurred, monitoring assessment during the occurrence of the disaster, and assessment after the disaster has occurred [23]. These disaster assessment methods are based on the actual assessment of historical disaster data; (2) The data used in the study are categorized using an indicator method [24,25], probability statistics, and scenario simulation [26,27], which differ for large, medium, and small disasters; (3) The causal relationship between multiple disasters is divided into multi-disaster superposition assessment [28,29] and disaster chain assessment [21,30]. The differences between the disasters in the multi-disaster overlay assessment method are represented by weights. The disaster chain assessment method emphasizes the causal relationship between disasters and investigates triggering disasters that occur in different time periods and spatial locations. In addition, some scholars have tried to conduct multi-disaster risk assessments at different scales, including a global scale [19], national scale [31], and regional or local scale [32,33].

Our review of the existing literature indicates that most disaster assessments in recent years are comprehensive analyses using indicators and data to establish index systems and functional models. The main difference between these evaluation indicators is that the criteria for determining the weight of each indicator are different. The existing weight determination methods mainly include principal component analysis, analytic hierarchy process, and entropy weight method. Among them, the analytic hierarchy process is subjective, and the weight value is susceptible to the randomness in the evaluation process and the subjective uncertainty of the evaluation expert and the ambiguity of the understanding. The entropy method and the principal component method are relatively objective, but the principal component method loses more information in the calculation process. Considering that the measurement units of various indicators for comprehensive disaster risk assessment are not uniform, in order to solve the homogenization problem of different quality indicators relatively objectively, this paper adopts the entropy method to determine the weight of different evaluation indicators. In addition, GIS provides a visual representation of the results. This study is based on existing research methods to determine urban multi-disaster risk. With reference to the existing studies, we develop an index system for urban disaster risk assessment. By using indicator weights, we can determine the comprehensive disaster risk. We apply the proposed research method to Guangzhou City to verify the feasibility of the method.

## 3. Materials and Methods 

### 3.1. Overview of the Study Area

**Figure 1:**
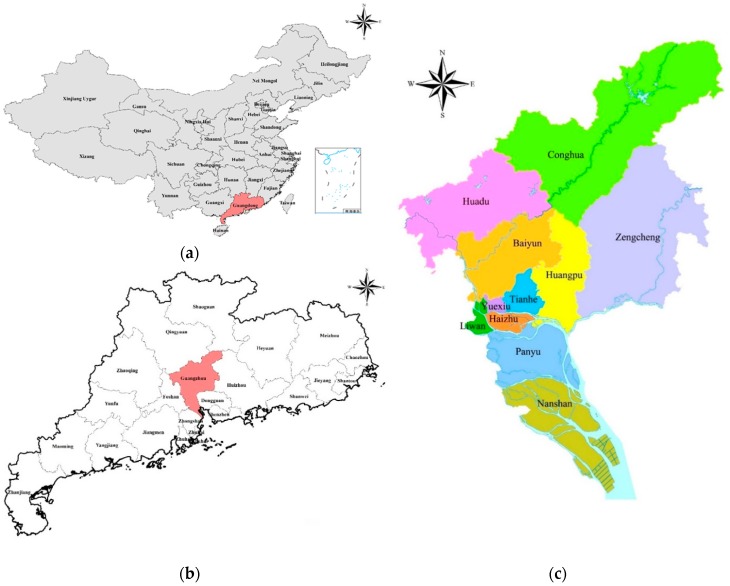
Location maps; (**a**) the location of Guangdong Province in China; (**b**) the location of Guangzhou in Guangdong Province; (**c**) the administrative division map of Guangzhou.

Guangzhou is located in the south-central part of Guangdong Province at the northern edge of the Pearl River Delta (Figure 1). Known as China’s “South Gate”, Guangzhou is the intersection of China’s most important high-speed railways and the South China Civil Aviation Transportation Center. Guangzhou City covers 7434.4 square kilometers and consists of 11 districts, namely Yuexiu, Liwan, Haizhu, Tianhe, Baiyun, Huangpu, Panyu, Huadu, Nansha, Zengcheng, and Conghua. In 2017, the population of Guangzhou reached 144.984 million with an urbanization rate of 86.14%. In 2017, the gross domestic product (GDP) of Guangzhou was 2105.315 billion CNY, and the per capita GDP was 150,678 CNY. Guangzhou is located in a subtropical maritime monsoon climate zone with abundant sunshine and rainfall. The annual average temperature in Guangzhou ranges from 21.4 °C to 21.9 °C and the annual average precipitation ranges from 1623.6 mm to 1899.8 mm. The terrain of Guangzhou is inclined from the northeast to the southwest. The north is dominated by mountains and hills, the central part is dominated by terraces, and the south part is dominated by plains. There are six dominant landforms in the city, including mountains, hills, mounds, terraces and plains. The rivers in Guangzhou run north to south and west to east and are part of the Pearl River system.

According to official statistics, Guangzhou is one of the most disaster-prone cities in China and is struck regularly by typhoons, storm surges, floods, lightning, geological disaster, fires, and other hazards, all of which have resulted in heavy loss of life and economic losses. A comprehensive assessment of the frequency of disasters, the damage caused by the disasters and the degree of danger indicates that the main disasters currently facing Guangzhou include floods, storm surges, earthquakes, geological disasters, and fires (Table 1). In this study, we consider these five disasters when assessing the disaster risk of Guangzhou City.

### 3.2. Methods for Identifying Major Disasters and Assessing Disaster Risk

An integrated disaster risk is the product of the interaction of subsystems in a disaster system. Any change of the subsystems will have an impact on the integrated disaster risk. The subsystems mentioned here are the various hazard factors. In a city disaster system in which multiple disasters coexist, a disaster group is comprised of a single disaster and the induced secondary or derivative disasters are intertwined to form a disaster chain. This highlights the comprehensiveness and complexity of the urban disaster system [34]. Therefore, a comprehensive perspective and method are needed for disaster risk analysis. The fuzzy evaluation method theory is derived from Fuzzy Maths [35]. The basic idea of this theory is to use the fuzzy linear transformation principle and the principle of maximum membership degree to consider the various factors related to the things being evaluated and make a reasonable comprehensive evaluation. The fuzzy comprehensive evaluation considers the multi-factor system with fuzzy nature and comprehensively applies various methods to evaluate it [35,36,37]. Urban disaster system is a fuzzy system composed of many factors of natural environment and socio-economic environment. In this paper, we conduct a reasonable and scientific assessment of urban disaster risk based on the basic steps of fuzzy comprehensive evaluation. Then, we think that the fuzzy comprehensive evaluation method is the most commonly used method based on the index system, which can better analyze the random ambiguity problem and is suitable for multi-index comprehensive evaluation. This method makes up for the shortcomings of adding the results of different disaster assessments in the traditional method. In this study, the multi-disaster disaster risk assessment method based on fuzzy comprehensive evaluation method consists of the following steps:

- *Identifying Major Disasters*. According to the frequency of disasters, the damage caused and the destructive magnitude of the disaster, we can identify the most common types of disasters in the city.
- *Establish a set of disaster risk factors U*. Be determining the frequency, economic losses, and degree of danger of major disasters, we can identify the major types of disasters in the study area and establish a set of disaster risk factors. The formula is as follows:
  (1) $$U=\left\{U_{1},U_{2},U_{3},\cdots ,U_{i}\right\}=\left\{U_{11},U_{12},U_{13},\cdots ,U_{ij},\right\},\;$$
- *Establish an evaluation set V*. In this study, we use the natural discontinuity grading method to partition the comprehensive evaluation values of the disaster risks into n grades. The higher the grade, the higher the disaster risk is. The natural discontinuity grading method is based on the natural grouping inherent in the data. The classification interval is identified, the similarity values are optimally grouped, and the differences between the classes are maximized. The formula is as follows:
  (2) $$V=\left\{V_{1},V_{2},V_{3},\cdots ,V_{N}\right\},N\in N*\;$$
- *Establishment of a single factor evaluation matrix*. We establish a fuzzy relationship that maps the disaster factor set to the evaluation set. The formula is as follows:
  (3) $$f:U\to F\left(V\right),\forall u_{i}\in U,\;$$
  (4) $$u_{i}|\to f\left(u_{i}\right)=\frac{r_{i1}}{v_{1}}+\frac{r_{i2}}{v_{2}}+\cdots +\frac{r_{im}}{v_{m}},\left(0\leq r_{ij}\leq 1,1\leq i\leq n,1\leq j\leq m\right),\;$$
  Then we obtain a fuzzy relationship matrix of a single factor:
  (5) $$R=\left[\begin{array}{ccc} r_{11} & r_{12} & \begin{array}{cc} \cdots & r_{1m} \end{array}\\ r_{21} & r_{22} & \begin{array}{cc} \cdots & r_{2m} \end{array}\\ \begin{array}{c} \vdots \\ r_{n1} \end{array} & \begin{array}{c} \vdots \\ r_{n2} \end{array} & \begin{array}{cc} \begin{array}{c} \vdots \\ \cdots \end{array} & \begin{array}{c} \vdots \\ r_{nm} \end{array} \end{array} \end{array}\right],\;$$
  Then (*U*, *V*, *R*) constitutes a model for comprehensive disaster risk assessment.
- *Calculation of indicator weight value*. In order to reduce the subjective influence on the weight determination, we use the entropy weight method to determine the weights of the evaluation indices. The improved model is as follows:
  The original data form the matrix *X*:
  (6) $$X=\left[\begin{array}{ccc} X_{11} & \cdots & X_{Im}\\ \vdots & \ldots & \vdots \\ X_{n1} & \cdots & X_{nm} \end{array}\right],$$
  By normalizing the raw data, we obtain a new matrix *Y*:
  (7) $$Y=\left[\begin{array}{ccc} Y_{11} & \cdots & Y_{Im}\\ \vdots & \ldots & \vdots \\ Y_{n1} & \cdots & Y_{nm} \end{array}\right],\;$$
  Then we derive the entropy value:
  (8) $$e_{j}=-k\sum_{t}\sum_{i}P_{ij}\ln P_{ij},\;$$
  (9) k=1/ln(t∗n), 
  (10) $$P_{ij}=Y_{ij}/\sum Y_{ij},\;$$
  The weight value *W_j_* is calculated based on the entropy value:
  (11) $$W_{j}=\frac{1-e^{j}}{\sum_{j=1}^{m}\left(1-e^{j}\right)},\;$$
  where *X_ij_* is an element in matrix *X*, *t* is the number of years, *m* is the number of indices, *n* is the number of samples and *e^j^* is the entropy value.
- *Comprehensive assessment of disaster risk*. We determine the weight values of the disasters using the historical statistics of the occurrence of each disaster. Based on the results of the different disaster risk assessments, we calculate the comprehensive disaster risk. The model is as follows:
  (12) $$B=\sum_{j=1}^{n}W_{j}*(\sum_{i=1}^{m}W_{i}F_{i}),\;$$
  where *n* represents the number of disaster species, *m* represents the number of indicators, *W_j_* represents the weight value of each disaster, and *W_i_* represents the weight value of each indicator.
- *Risk classification and coping strategies.* Based on the comprehensive disaster risk assessment, we can grade the results of the comprehensive assessment and obtain different disaster risk zones. In this paper, we mainly use the Natural Breaks method to classify disaster risk levels. The Natural Breaks method is based on the natural grouping inherent in the data. The classification interval is identified, the similarity values can be optimally grouped, and the differences between the classes can be maximized.

### 3.3. Index System

Disaster risk assessment is a key to the city’s major disasters, based on risk theory and methods, to analyze the possibility and consequences of the occurrence of the damage, and to provide a basis for urban disaster prevention and mitigation work. In general, disaster risk assessment content mainly includes risk, environment, vulnerability and disaster prevention. The risk mainly reflects the intensity and frequency of disaster occurrence. The environment of the disaster mainly includes the geographical environment, geological conditions and climatic conditions for the disaster. Vulnerability assessment is based on the perspective of the affected population and economic losses. Under the same conditions of the urban disaster prevention ability and disaster level, the higher the population density is, the more likely it is to cause casualties. And the higher the economic level is, the more serious the economic losses will be. The population density and economic development level of a certain area of the city can reflect the vulnerability of the region. The disaster prevention capability is judged from the construction and distribution of evacuation sites, urban rescue capabilities, and emergency management capabilities. The smaller the disaster prevention capability is, the higher the disaster risk is. In addition, there are special evaluation factors for different disasters. For example, the urban drainage system has an impact on the disaster prevention capability of the guilt, and the urban fire protection capability has an impact on the fire prevention capability. We need to adjust specific indicator factors in combination with different disasters. We established an index system for evaluating the comprehensive disaster risk of Guangzhou by referring to the comprehensive risk assessment index system proposed by different scholars [38,39,40,41] combined with the characteristics of the disaster risk in Guangzhou (Table 2).

## 4. Results

By evaluating the previous occurrence of major disasters (Section 3), we determined that the types of disasters with the greatest impact on Guangzhou were floods, storm surges, earthquakes, geological disasters, and fires. Using the current data and the calculation formulas, we evaluated the different disaster risks separately and then obtained the comprehensive assessment of disaster risk in Guangzhou.

### 4.1. Assessment of Flood Risk

**Figure 2:**
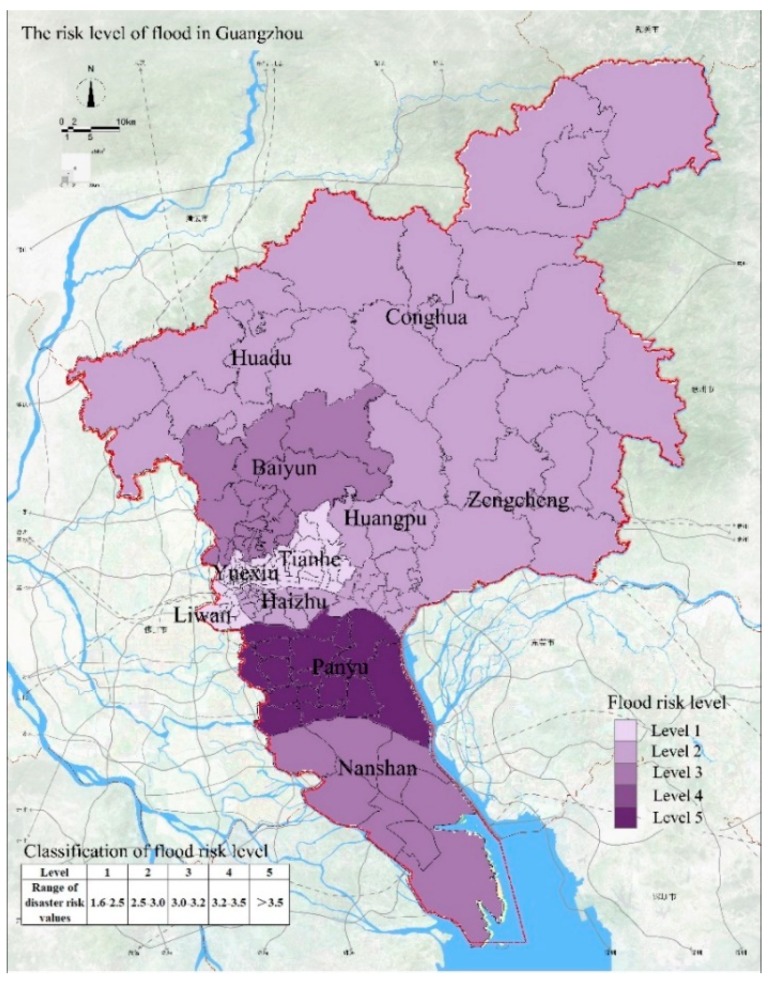
The risk levels of flood in Guangzhou.

Guangzhou is an area that experiences frequent natural disasters. According to the relevant statistics, from 1949 to 2015, there were 21 floods in Guangzhou and seven floods in the basin. The basin floods caused different degrees of loss and threatened the development of Guangzhou. Based on the indicators and weight values of flood disasters, we obtain the assessment results of flood disaster risk in Guangzhou (see as Figure 2). We determined that the area with the highest flood risk is Panyu District and the areas with the lowest risks are Yuexiu District and Liwan District. The flood risk level in Panyu District is 5. We believe that this attributed to the low altitude in Panyu District, which results in excessive runoff. In addition, because Panyu District is close to the Pearl River estuary, the density of the water network is relatively high and the area is susceptible to water accumulation. Therefore, Panyu District has the highest flood disaster risk. The flood risk level of Nansha District is 4 and this area is also affected by the offshore estuary. The flood risk level in Huangpu District and Baiyun District is 3 and this risk level is attributed to the low density of the drainage network and the slow drainage of rainwater. The risk level of floods in Huadu District, Haizhu District, Zengcheng District, and Conghua District is 2, representing a low probability of flood disaster. The flood risk level is 1 for the Liwan District, Yuexiu District, and Tianhe District, indicating that these areas are less likely to be affected by flood disasters.

### 4.2. Assessment of Storm Surge Risk

**Figure 3:**
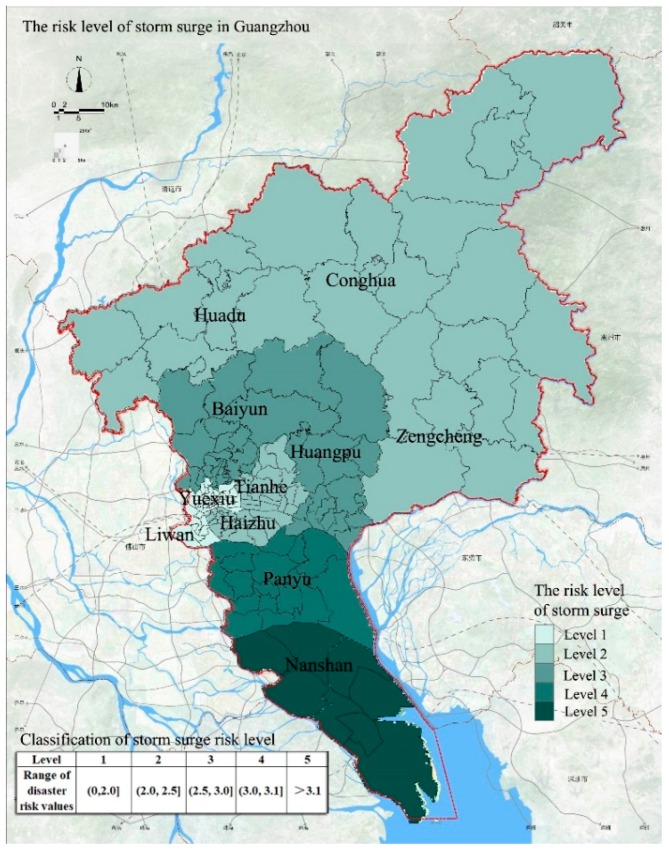
The risk levels of storm surge in Guangzhou.

Storm surge refers to an abnormal rise and fall of the sea level due to severe atmospheric disturbances such as strong winds and sudden changes in air pressure. Because of the proximity to the estuary, the area adjacent to the estuary in Guangzhou is affected by the storm surge. At the same time, as the river system of Guangzhou is connected to the ocean, the rise of sea level will also affect the water level of urban rivers. The frequency and intensity of tropical cyclones in Guangdong Province are the highest in China. On average, 3.54 tropical cyclones occur in Guangdong Province each year, accounting for 37% of the national occurrence, and up to seven cyclones have occurred in the past. Storm surges caused by strong tropical cyclones often lead to extreme weather events such as heavy rainfall, strong winds, large waves, and seawater intrusion. These events cause large numbers of casualties and property damage. The comprehensive assessment of the storm surge risk in Guangzhou is shown in Figure 3. The area with the highest risk level of storm surge disasters in Guangzhou is Nansha District, whereas Yuexiu District and Liwan District have the lowest risk level. The storm surge risk level in Nansha District is 5. We attribute this level to the low elevation of the Nansha District that results in excessive surface runoff. When a storm surge is followed by heavy rainfall, water accumulation occurs. In addition, Nansha District is close to the estuary of the Pearl River and the area often is submerged due to high tides caused by the storm surge. Moreover, the density of the water network in the area is relatively high, making the area susceptible to the impact of seawater intrusion caused by storm surges. As a result, the risk of storm surge disasters is highest in Nansha District. The storm surge risk level in Panyu District is 4 and this is also attributed to the proximity to the estuary, resulting in a higher risk. The storm surge risk level in Baiyun District and Huangpu District is 3; these areas have relatively low drainage capacity. The storm surge risk level of Huadu District, Conghua District, Zengcheng District, Tianhe District, and Haizhu District is 2, indicating a low probability of storm surge disasters. The storm surge risk level in Liwan District and Yuexiu District is 1, indicating that these areas are not affected by storm surge disasters.

### 4.3. Assessment of Earthquake Risk

According to statistics, 20 earthquakes of magnitude 4 or higher (including 19 earthquakes with magnitude 4 or higher and one earthquake with magnitude 5) and six earthquakes with a seismic intensity equal to or greater than 5 have occurred in Guangzhou. Guangzhou City is located in the Pearl River Delta Earthquake Key Monitoring and Defense Zone and there are many fault zones in the urban area. We assessed the risk of earthquake disasters from three aspects: natural disaster intensity, seismic vulnerability, and seismic response capability. In our article, when we discuss the natural disaster intensity of an earthquake in a certain area, we mainly consider the following three points: (1) Whether there is a distribution of seismic fault zones in the area, and the distance from the earthquake fault zone; (2) Has there been an earthquake in the area? (3) Earthquake fortification intensity in the area. According to the specific situation of Guangzhou, although there are several small fault zones distributed in the city area, the geological tectonic movements are not frequent, and no earthquake has occurred in the past 100 years. At the same time, the seismic fortification intensity in most parts of Guangzhou is VII. Due to the uncertainty of the location of an earthquake, the natural disaster intensity is difficult to determine in a small area. Therefore, for the assessment of earthquake risk, we assume that the natural disaster intensity is the same in all areas of Guangzhou. Based on this premise, we discuss the relative risk of earthquake risk. Generally, we assume that the smaller the earthquake vulnerability index in a region, the greater the seismic response capability is and the lower the earthquake risk is in this region [8,9].

**Figure 4:**
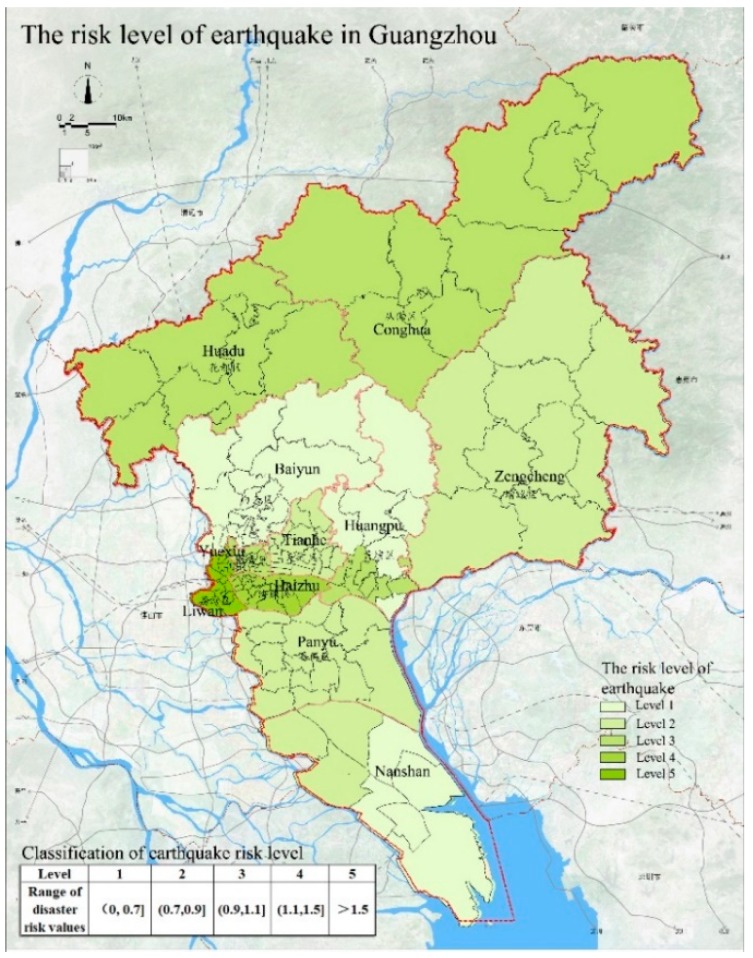
The risk levels of earthquake in Guangzhou.

The assessment results of the earthquake risk in Guangzhou are shown in Figure 4. We divide the earthquake risk into five levels. The higher the risk level, the higher the earthquake risk is. The earthquake risk level in Liwan District and Yuexiu District is 5 and the risk level is highest in these two regions. This is attributed to the high population density and building density in the regions. We believe that under the same conditions of urban prevention and earthquake disaster level, the higher the population density is, the more likely it is to cause casualties. And the higher the economic level is, the more serious the economic losses will be. In addition, due to the low density of public spaces and rescue capabilities of these regions, the seismic response capability is weak. The earthquake risk level is 4 for the Haizhu District, Huadu District, and Conghua District; this risk level is related to the low quality of the buildings and the geological conditions in the area. The earthquake risk level in Tianhe District is 3 and is also affected by the low quality of the buildings in the area. The earthquake risk level is 2 for the Panyu District, Zengcheng District, and Huangpu District, indicating a relatively low probability of earthquake disasters in these areas. The earthquake risk level of Baiyun District and Nansha District is 1, indicating that the risk is lowest. Because the population density and building density are not high in these two regions and the seismic response capability is relatively good, these two regions are not much affected by earthquakes. The analysis of the earthquake disaster risk in Guangzhou indicates that the low quality of the buildings is the most important factor affecting the risk level of earthquakes in Guangzhou.

### 4.4. Assessment of Geological Disaster

**Figure 5:**
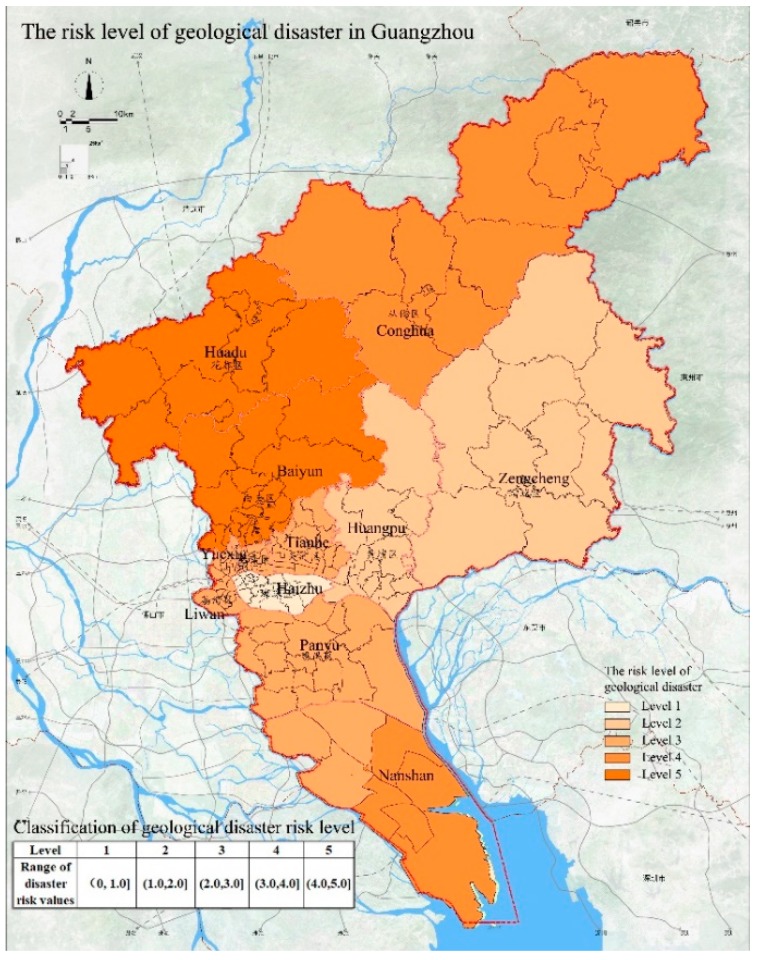
The risk levels of geological disaster in Guangzhou.

The topographic and geological conditions of Guangzhou City are relatively complex and there are many faults. In addition, due to natural environmental conditions and human engineering activities, geological disasters often occur in Guangzhou. The types of geological disasters that have occurred in Guangzhou include collapses, landslides, debris flows, ground subsidence, land subsidence, foundation settlement, and soil erosion. Among the 190 geological disasters that have occurred in Guangzhou, 39 were induced by natural factors, accounting for 20.5%, and 151 were induced by human factors, accounting for 79.5%. It is evident that human factors are the main reasons for geological disasters. The distribution of geological disasters in Guangzhou is uneven and most have occurred in Huadu District, Zengcheng District, and Conghua District (Table 3). The risk levels of geological disasters in Guangzhou are shown in Figure 5. The risk level is highest (5) in Baiyun District. This is attributed to a large number of geological hazard locations in the area (Table 3) and in addition, the area is close to many faults, which may the source of many disasters in Baiyun District. The risk level of Huadu District, Conghua District, and Nansha District is 4 and is also the result of the proximity to the faults and geological disaster locations. The risk level in Panyu District, Yuexiu District, Tianhe District, and Liwan District is 3, representing a medium risk level, which is also related to the geological disaster locations in these areas. The risk level in Huangpu District and Zengcheng District is 2. There were fewer geological disasters in these two regions, resulting in a lower risk level. The risk level of geological disasters in Haizhu District is 1, indicating that this area is not much affected by geological disasters.

### 4.5. Assessment of Fire Disaster

**Figure 6:**
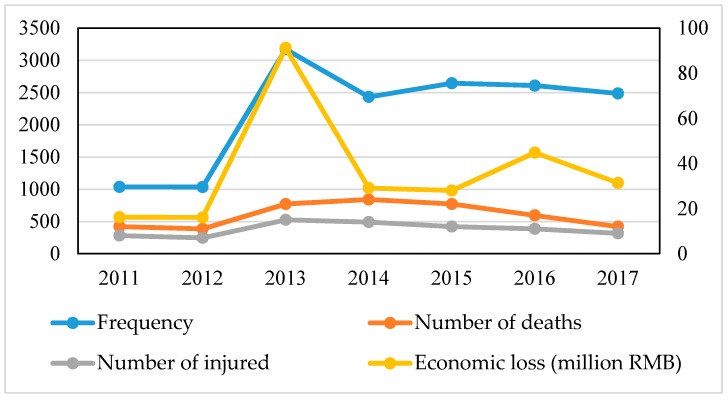
Fire statistics in Guangzhou from 2011 to 2017.

**Figure 7:**
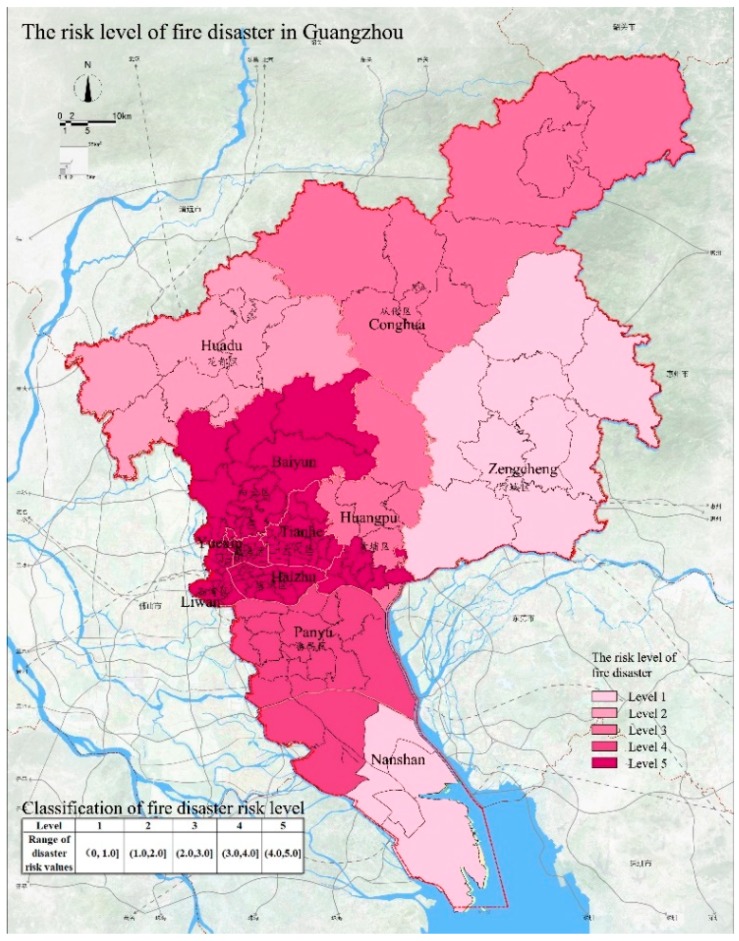
The risk levels of fire disaster in Guangzhou.

The frequency of fire disasters in Guangzhou is high and the losses are often serious. The fire statistics from 2011 to 2017 (Figure 6) indicate that the number of fires in Guangzhou has remained generally stable, but a large number of fires occurred in 2013. When assessing the risk of urban fires, we need to consider the three aspects of the fire hazard, the effect on people, and the status of the urban fire protection system. The risk assessment results of fire disasters in Guangzhou are shown in Figure 7. The fire risk level of the Liwan, Yuexiu, Tianhe, and Haizhu Districts is 5 and the high risk level is mainly attributed to the generally poor quality of the buildings in these areas. The fire risk level is 4 for the Panyu District, Baiyun District, and Huangpu District; these areas are also affected by poor fire protection conditions. The Conghua District has a fire risk level of 3 and this area is also affected by the fire protection facilities but to a lesser degree. Huadu District has a fire risk level of 2, which indicates a low probability of fire. Nansha District and Zengcheng District have a fire risk level of 1, indicating the lowest risk of fire disaster. This is related to the low population density and low building density and the relatively good fire response capability of the two regions.

### 4.6. Comprehensive Assessment of Disaster Risk in Guangzhou

The weights of the disasters were calculated (Table 1) and show that storm surges and floods are the two most common disasters in Guangzhou. Their weight values are 0.33 and 0.27. The impact of fire disasters and geological disasters ranks second with weight values of 0.2 and 0.13. Earthquake disasters have the smallest probability and the weight value is 0.07.

**Figure 8:**
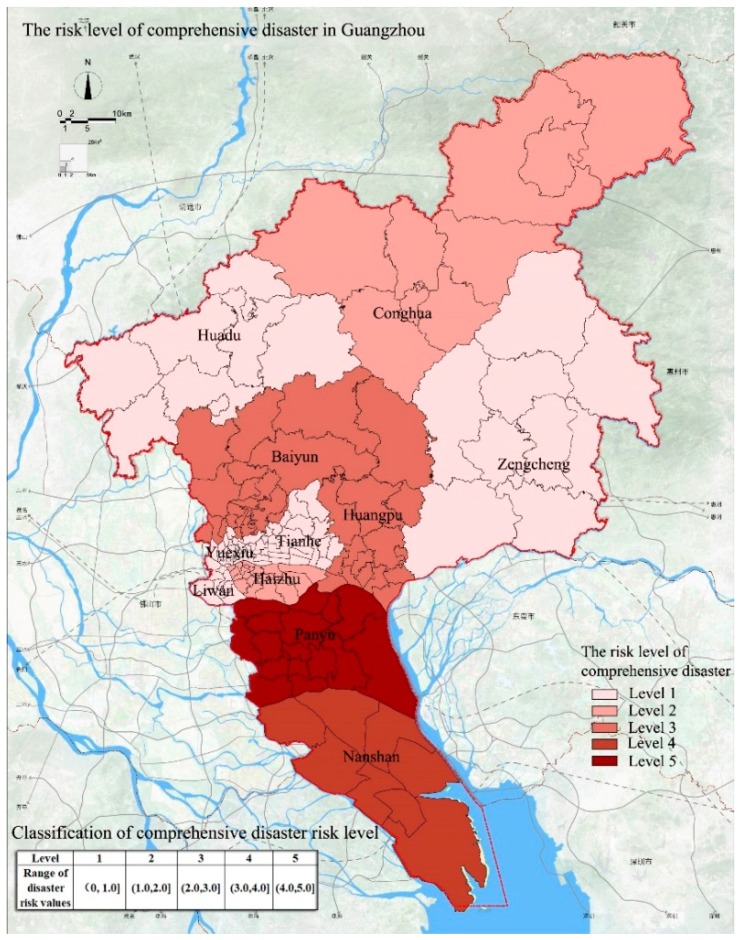
The risk levels of comprehensive disaster in Guangzhou.

Using the weight values and the assessment results of the individual disasters in Guangzhou, we obtain the assessment results of the comprehensive disaster risk (Figure 8). The results show that the area with the highest risk level of 5 is Panyu District; this high risk level is attributed to the high risk of flood disasters, storm surge disasters, and fire disasters. The comprehensive disaster risk level of Nansha District is 4. One reason for this risk level is the proximity to the estuary, which results in the highest risk of storm surge disasters in this area. Another reason is that the risk of geological disasters is high in this area. The comprehensive disaster risk level of Huangpu District and Baiyun District is 3 and these areas are mainly affected by fire disasters and geological disasters. The comprehensive disaster risk level of Haizhu District and Conghua District is 2; Haizhu District is mainly affected by fires and Conghua District is affected by geological disasters. The comprehensive disaster risk levels of Huadu District, Zengcheng District, Liwan District, Yuexiu District, and Tianhe District are 1, indicating that these areas are relatively safe.

## 5. Discussion

The disaster risk assessment facilitates the identification of the risk environment in the city. The disaster risk assessment results can also be used to guide safe urban development and construction. During urban planning and construction, the adaptability of cities to disaster risks should focus on two aspects: (1) the spatial layout and development direction of the city should be adjusted to minimize the risk of disasters and enhance the city’s security; (2) Urban disaster risk should be evaluated based on the disaster risk assessment results and we should plan for various types of refuge facilities and shelters for different disaster risk levels to improve the city’s ability to respond to disasters.

The results of the comprehensive disaster risk classification in Guangzhou (Figure 7) demonstrate that the disaster risk is significantly higher in southern Guangzhou than in the north, which is related to the proximity to the estuaries and the number of geological disasters that occurred in the past in the southern region. Therefore, for future planning and construction in Guangzhou City, the focus of urban development should be the northern region and the direction of urban development should be extended to the central and northern regions to improve the safety of the city. In addition, considering the comprehensive disaster prevention requirements and the characteristics of the disaster chain, we need to evaluate the suitability of different areas for evacuation sites and evacuation facilities to optimize the spatial layout of the evacuation sites [9] and reach the goal of improving the city’s ability to cope with disasters. Specifically, we propose different requirements for areas with different disaster risk levels in Guangzhou:

- *Highest disaster risk zone.* The terrain of this area is relatively complex, and there are many geological disaster locations. Because of the proximity to the estuary, the number of storm surge disasters and flood disasters is high, resulting in a higher disaster risk in this area. In addition, the vegetation cover in this area is high and the ecosystem stability is low. This makes the area extremely vulnerable to human activities and has considerable influence on the productivity and construction in the city. Therefore, the area is a key zone that should be protected from urban development and is not suitable for further urban development. In the process of urbanization, we should consider safety, urban productivity, and human life. We also need to focus on green construction and the reduction of the population density and urban building density. In addition, the relevant departments should be required to formulate policies, laws, and regulations to strictly protect the areas and prohibit high-intensity development and construction.
- *Higher disaster risk zone.* This zone occurs mainly in the southern region, which is close to the highest risk area. Because of the proximity to the estuary, storm surges and internal disasters in the area are more frequent. The land use intensity and population density in the southern region are relatively low, which is conducive to the development of safe cities. For future city development, it is necessary to avoid urban development in the southern region. Local government departments must formulate policies and laws to strictly protect and prohibit high-intensity development and construction. In addition, considering that the area is adjacent to the estuary, it is necessary to create a green belt as a buffer zone to minimize the impact of disasters such as typhoons.
- *Medium disaster risk zone.* Judging by the current status of development and construction, this area is a region with high land-use intensity and low population density in Guangzhou. This area is close to the core area and has certain location advantages. This area should be reserved for urban construction and urban growth to integrate urban development and ecological protection.
- *Lower disaster risk zone.* The region has low disaster risk and can be used for urban development. Judging by the current status of development and construction, the region has also high land-use intensity and low population density. This is a result of the mountainous terrain in this region, making less land available for development and utilization. In the long-term, this area should be reserved for future development.
- *Lowest disaster risk zone.* The region is best suited as an area for urban construction and development. Judging by the current status of urban construction, this region has the highest land-use intensity and highest population density in Guangzhou. The area can be used as urban reserve land after considering engineering measures and environmental protection but it is necessary to avoid excessive development.

## 6. Conclusions

In this study, we proposed a method for urban multi-disaster risk assessment that integrated aspects of existing disaster risk assessment methods. The multi-hazard risk assessment method includes the identification and categorization of urban disasters, the assessment of individual disaster risks, the assessment of integrated urban disaster risks, the division of the urban comprehensive disaster risk levels, and the determination of coping strategies. For the identification and categorization of urban disasters, we considered the frequency of the disasters, the number of casualties, and the economic losses. For the single disaster risk assessment, we developed an index system for evaluating each disaster risk and calculated the weights of the indices using the entropy weight method. For the urban comprehensive disaster risk assessment, we first calculated the weights based on the frequency and amount of damage caused by the disasters. The assessment results of the urban comprehensive disaster risks were categorized into several levels and response strategies were proposed based on the locations and risk levels. In order to verify the applicability of the proposed method, we used Guangzhou as an example to conduct empirical research. The following conclusions were obtained:

- Guangzhou is a city affected by multiple disasters. The comprehensive assessment of the frequency of disasters, the damage caused by disasters and the degree of danger indicates that the main disasters currently facing Guangzhou are floods, storm surges, earthquakes, geological disasters, and fire disaster.
- The risk of flood disasters is higher in the southern region of Guangzhou than in the northern and central regions; this is related to the close proximity of the southern region to the estuary and lake network. Storm surge disasters in Guangzhou exhibit the same spatial distribution as flood disasters. The risk of storm surge disasters is much higher in the southern region than in the central and northern regions. We believe this is also attributed to the proximity to the estuary and the dense lake network. The central and northern regions in Guangzhou have high population density and poor building quality, which results in high earthquake risk in these two regions. In the eastern and southern regions, the risk of earthquake disasters is low due to the low population density and low building density. Geological disasters are relatively high due to the large distribution of historic geological disasters and the complex terrain in the northern and southern regions. The risk of geological disasters is relatively low in the central region. The most important factors affecting the frequency of fire disasters in Guangzhou are the population density and building density. The central region has a relatively high population density and building density; therefore, the fire risk is relatively high.
- The comprehensive disaster risk results indicate that storm surges and floods pose the greatest threat to Guangzhou, followed by fire, geological disasters, and earthquakes. The comprehensive risk is highest in the southern region and relatively low in the central region and the northern region. The following coping strategies are proposed. It is necessary to reduce the amount of construction and development in the southern region with the high disaster risk and appropriately guide the urban development in the northern region.

Urban disaster risk assessment is a basic and important component of urban safety development and construction. Conducting disaster prevention and mitigation on the basis of urban disaster risk assessment requires an understanding of the relationship between the city and the natural environment. This enhances the city’s ability to withstand various types of disasters and achieves the development of safe cities. Urban disasters are characterized by complexity and diversity and risk assessment of urban disasters is a challenging task. In future research, we will focus on the locations and layouts of urban emergency shelters and on urban public safety planning and emergency management.

## Figures and Tables

**Table 1:** The most common types of disasters in Guangzhou.

Disaster	Disaster Frequency	Disaster loss
Typhoon	32 typhoons landed near Guangzhou from 1988 to 2017.	Disaster losses are mainly reflected in storm surge disasters and flood disasters.
Storm surge	3.2 per year on average	In the past 50 years, the losses caused by storm surges in Guangzhou has reached 1.68 billion CNY.
Flood	20 per year on average	In the past 50 years, the losses caused by floods have been nearly 2.35 billion CNY, and the average annual losses accounted for 0.3% of Guangzhou’s GDP.
Geological disaster	190 geological disasters occurred from 1992 to 2015	From 1992 to 2015, 106 people were killed, 10 people were declared missing and 426 people were injured. The direct economic loss was 580 million CNY.
Earthquake	20 earthquakes with a magnitude of more than 4 of the Richter scale occurred	No earthquake has occurred in recent years, but there is still the possibility of an earthquake.
Fire disaster	2201 per year on average from 2011 to 2017	From 2011 to 2017, 17 people were killed per year, 11 people were injured per year, and the direct economic losses reached 37 million yuan per year.
Dangerous chemicals and explosives	1 to 2 times a year since 2005	The damage caused is different.
Public health security incidents	Potential disaster	Potential disaster

Source: constructed by authors, using data from the website of Guangzhou government.

**Table 2:** Index system for evaluating the comprehensive disaster risk of Guangzhou.

Disaster	Weight	First Grade Index	Basic Grade Index	Weight
Flood	0.27	Susceptibility to disaster factors	Precipitation	0.1
			Flooded area	0.25
			River distribution	0.1
			Guilty disaster point	0.2
		Sensitivity to the environment	Terrain	0.05
			Land type	0.05
		Vulnerability of the disaster-bearing body	Population density	0.05
			GDP per district	0.05
		Disaster prevention and resilience	Drainage pipe coverage	0.15
Storm surge	0.33	Susceptibility to disaster factors	Precipitation	0.1
			Storm surge flooded area	0.25
			River and reservoir distribution	0.1
			Guilty disaster point	0.15
		Sensitivity to the environment	Terrain	0.075
			Land type	0.075
		Vulnerability of the disaster-bearing body	Population density	0.05
			GDP per district	0.05
		Disaster prevention and resilience	Drainage pipe coverage	0.15
Earthquake	0.07	Natural disaster intensity	Seismic geological environment	0.09
			Seismic activity	0.09
			seismic intensity	0.12
		Seismic vulnerability	Building seismic capacity	0.23
			Population density	0.12
			GDP per district	0.06
		Earthquake response capability	Public space density	0.09
			Rescue ability	0.05
			Ambulance ability	0.11
			The perfection of emergency system	0.05
Geological disaster	0.13	——	Disaster point distribution	0.4
			Topography	0.3
			Distribution of human engineering activities	0.1
			Geological structure	0.2
Fire disaster	0.20	City characteristics	Population density	0.22
			GDP per district	0.18
			The quantity number of crowded places	0.06
		Architectural characteristics	Building age	0.12
			The quantity number of high-rise buildings per district	0.07
		Fire load	The quantity number of flammable and explosive goods storage areas per district	0.10
		Firefighting ability	The quantity number of firefighters per 10,000 people	0.15
			Fire service coverage	0.10

Source: The economic, population and other data involved in this paper are from Guangzhou Statistical Yearbook and Guangzhou City Economic Census Data. Data such as precipitation, floods, earthquakes and typhoons come from the Guangzhou Meteorological Bureau, the Oceanic Administration, the Earthquake Administration and the Emergency Office. The relevant data on land and topography comes from the Guangzhou Municipal Bureau of Land and Resources and the Earthquake Administration. The relevant data of the road network and other municipalities come from the Guangzhou Municipal Transportation Bureau, the Housing Construction Bureau and the Statistical Yearbook.

**Table 3:** Distribution of geological disasters in Guangzhou.

District	Collapse	Landslide	Debris Flow	Ground Collapse	Ground Subsidence	Soft Foundation Subsidence	Total	Percentage%
Central area ^1^	2	42	4	4	1		53	27.89
Panyu	2	1					3	1.58
Nansha	18	15	1	13			47	24.74
Huadu	3	7	1	27			38	20.0
Zengcheng	4	1					5	2.63
Conghua	8	76	1	11	12	5	44	23.16
Total	37	73	7	55	13	5	190	100

^1^ Guangzhou’s Central area mainly includes Yuexiu District, Liwan District, Haizhu District, Tianhe District, Huangpu District and Baiyun District.
